# Thrombocytosis and hyperfibrinogenemia are predictive factors of clinical outcomes in high-grade serous ovarian cancer patients

**DOI:** 10.1186/s12885-016-2070-2

**Published:** 2016-01-27

**Authors:** Zheng Feng, Hao Wen, Rui Bi, Yachen Duan, Wentao Yang, Xiaohua Wu

**Affiliations:** Department of Gynecological Oncology, Fudan University Shanghai Cancer Center, 270 Dong-an Road, Shanghai, 200032 China; Department of Oncology, Shanghai Medical College, Fudan University, Shanghai, 200032 China; Department of Pathology, Fudan University Shanghai Cancer Center, Shanghai, 200032 China

**Keywords:** Ovarian cancer, Platelet, Fibrinogen, Tumor burden, Prognosis

## Abstract

**Background:**

Over 20 % of ovarian cancer patients have preoperative thrombocytosis or hyperfibrinogenemia. We aimed to demonstrate the clinical and prognostic significance of thrombocytosis and hyperfibrinogenemia in high-grade serous ovarian cancer (HGSC).

**Methods:**

We retrospectively investigated HGSC patients who underwent primary staging or debulking surgery between April 2005 and June 2013 in our institution. None of these patients had received neoadjuvant chemotherapy. Data, including age, performance status, FIGO stage, serum CA125, platelet count, fibrinogen level, and surgical residual disease, were collected. Thrombocytosis was defined as a platelet count greater than 450 × 10^9^/L, and hyperfibrinogenemia was defined as a fibrinogen level higher than 4.00 g/L. Progression-free survival (PFS) and overall survival (OS) were analyzed with the Kaplan-Meier method and log-rank tests for univariate analyses. For the multivariate analyses, Cox regression analysis was used to evaluate the effects of the prognostic factors, which are expressed as hazard ratios (HRs).

**Results:**

A total of 875 consecutive HGSC patients were identified. The median follow-up time was 29 (1–115) months. The median (interquartile range, IQR) preoperative platelet count was 301 (235–383) × 10^9^/L, and 121 (13.8 %) women had thrombocytosis. The median (IQR) preoperative fibrinogen level was 3.85 (3.19–4.45) g/L, and 332 (45.9 %) of the patients had hyperfibrinogenemia. Both preoperative thrombocytosis and hyperfibrinogenemia were associated with an advanced FIGO stage (*p* = 0.008 and <0.001, respectively), an increased CA125 level (*p* = 0.004 and 0.001, respectively), more extensive ascites (*p* < 0.001 and <0.001, respectively), more extensive residual disease (*p* < 0.001 and <0.001, respectively) and chemosensitivity (*p* = 0.043 and <0.001, respectively). In the univariate analyses, hyperfibrinogenemia was associated with reduced PFS (*p* < 0.001) and OS (*p* < 0.001). However, thrombocytosis was not found to be a potential predictor of PFS (*P* = 0.098) or OS (*p* = 0.894). In the multivariate analyses, hyperfibrinogenemia was an independent predictor of OS (*p* = 0.014) but not PFS (*p* = 0.062).

**Conclusion:**

Preoperative thrombocytosis and hyperfibrinogenemia reflected tumor burden to some extent and thus influenced treatment outcomes, and the fibrinogen level was found to be useful as a prognostic predictor in the HGSC patients.

## Background

Ovarian cancer is the seventh most commonly diagnosed cancer and the eighth leading cause of cancer death among females worldwide. There were 238,700 estimated new cases and 151,900 deaths in 2012 [1]. Cancer patients often present in a prothrombotic state. Approximately 20 to 30 % of ovarian cancer patients have preoperative thrombocytosis [2–4], and approximately 40 % of patients have preoperative hyperfibrinogenemia [5, 6].

Serologic coagulation parameters, including platelet count and fibrinogen level, have emerged as potential preoperative predictors of clinical outcomes [3, 5, 7]. Several studies have shown that either thrombocytosis or hyperfibrinogenemia is associated with an advanced FIGO stage, more extensive residual disease, and poorer prognoses in ovarian cancer patients [2–4]. However, other studies have failed to draw similar conclusions [8, 9]. The data are still insufficient to define the clinical and prognostic significance of platelet count and plasma fibrinogen level in ovarian cancer.

Furthermore, previous studies have shown that epithelial ovarian cancer is not a single disease but a group of heterogeneous tumors based on distinctive morphologic and molecular genetic features [10]. Previous research has failed to individually evaluate the clinical and prognostic values of platelet counts and fibrinogen levels according to histologic type. Because the vast majority of ovarian carcinomas are HGSC, the purpose of our study was to investigate the clinical and prognostic significance of the preoperative platelet count and fibrinogen level in a large mono-institutional study of Chinese patients with HGSC.

## Methods

### Clinical data

The clinical data were collected retrospectively from women who underwent primary staging or debulking surgery for HGSC between April 2005 and June 2013 at Fudan University Shanghai Cancer Center. This study was approved by the ethics committee of Fudan University Shanghai Cancer Center. All participants had provided written informed consent form. Patients were excluded if they had received neoadjuvant therapy, had been treated for recurrent disease, or were found to have other histological diagnoses on pathological review.

Clinical and pathological data were obtained from the medical records, cancer registries, and pathology reports. Patient characteristics including age, FIGO stage, preoperative laboratory data (CA125 level, platelet count, fibrinogen level), presence of ascites, surgical outcomes (R0, R1 *vs* R2), date of surgery, date of progression or recurrence, date of last follow-up, and the patient’s disease status at last contact, were collected.

The histological diagnoses were based on the WHO criteria, and all microscopic slides were reviewed by two experienced gynecologic pathologists. Eight hundred seventy-five consecutive patients were identified, and all of the patients were followed up until December 31st, 2014.

Thrombocytosis was defined as platelet count greater than 450 × 10^9^/L. Hyperfibrinogenemia was defined as fibrinogen level greater than 4.00 g/L. R0 was defined as the absence of macroscopic residual disease (RD) after surgery. R1 was defined as a maximal diameter of the macroscopic residual disease after cytoreduction of <1 cm. R2 was defined as a maximal diameter of residual disease of ≥1 cm. The optimal surgery was defined as the combination of R0 and R1. Chemosensitivity was defined as a time interval of 6 months or longer between the completion of platinum-based chemotherapy and the detection of relapse. Chemoresistance was defined as disease progression during adjuvant chemotherapy or within the 6-month interval between the completion of platinum-based chemotherapy and the detection of relapse.

Progression-free survival (PFS) was defined as the time interval from the date of primary surgery to the date of disease progression or recurrence. Overall survival (OS) was defined as the time interval from the date of the primary surgery to the date of death or the last follow-up.

### Statistical analyses

SPSS statistical software (version 21.0, SPSS, IBM Inc, New York, USA) was used for the statistical analyses. Descriptive statistics were used for the demographic data and are summarized as the means with the standard deviations (SD), the medians with the interquartile ranges (IQRs) or ranges, or the frequencies with the percentages. The categorical data were compared with chi-square or Fisher’s exact tests as appropriate. The PFS and OS were analyzed with the Kaplan-Meier method, and log-rank tests were used in the univariate analyses. For the multivariate analyses, Cox regression analysis was used to evaluate the effects of the prognostic factors, which are expressed as hazard ratios (HRs). *P* < 0.05 was considered statistically significant, and all reported *P* values were 2-sided.

## Results

### Patient characteristics and their correlations with thrombocytosis and hyperfibrinogenemia

The patient characteristics are shown in Table 1. Among the 875 patients, 874 (99.9 %) had documented preoperative platelet counts and 724 (82.7 %) had documented fibrinogen levels. The median (IQR) platelet count was 3.85 (3.19–4.45) × 10^9^/L, and 121 (13.8 %) women had preoperative thrombocytosis. The median (IQR) fibrinogen level was 3.85 (3.19–4.45) g/L, and 332 (45.9 %) had preoperative hyperfibrinogenemia. Seventy-seven patients had coexisting thrombocytosis and hyperfibrinogenemia. Seventy-five (8.6 %) patients were early stage (I-II) and 800 (91.4 %) were advanced stage (III-IV).

**Table 1:** Patient Characteristics (*n* = 875)

Age at diagnosis, median (range), years		56 (30–90)	
Follow-up time, median (range), months		29 (1–115)	
Menopausal status	Postmenopausal	602	68.8 %
	Premenopausal	273	31.2 %
Vital status	Died	345	39.4 %
	Alive	448	51.2 %
	Censored	82	9.4 %
Family history	Yes	230	26.3 %
	No	643	73.7 %
CA125 level	<500 U/ml	193	22.6 %
	≥500 U/ml	662	77.4 %
Ascites	No	99	11.3 %
	<500 ml	146	16.7 %
	≥500 ml	629	72.0 %
FIGO Stage	Early (I, II)	75	8.6 %
	Advanced (III, IV)	800	91.4 %
Cytoreduction	R0	272	31.1 %
	R1	434	49.6 %
	R2	169	19.3 %
Chemosensitivity	Yes	568	66.9 %
	No	237	27.9 %
	NA	44	5.2 %
Platelet count (median, IQR)(×10^9^/L)		301 (235–383)	
Thrombocytosis	Yes	121	13.8 %
	No	753	86.2 %
Fibrinogen level (median, IQR)(g/L)		3.85 (3.19–4.45)	
Hyperfibrinogenemia	Yes	332	45.9 %
	No	392	54.1 %

The correlations of the clinical characteristics with preoperative thrombocytosis and hyperfibrinogenemia are summarized in Tables 2 and 3, respectively. Both preoperative thrombocytosis and hyperfibrinogenemia were associated with an advanced FIGO stage (*p* = 0.008 and <0.001, respectively), an increased CA125 level (*p* = 0.004 and 0.001, respectively) and more extensive ascites (*p* < 0.001 and <0.001, respectively). There were no significant correlations of age with thrombocytosis or hyperfibrinogenemia (*p* = 0.493 and 0.117, respectively).

**Table 2:** Clinicopathologic parameters of the patients and thrombocytosis

Parameters		Thrombocytosis		*P*-value
		Yes	No	
Age	<56(427)	63(14.8 %)	364(85.2 %)	0.493
	≥56(447)	58(13.0 %)	389(87.0 %)	
FIGO Stage	Early(74)	3(4.1 %)	71(95.9 %)	0.008
	Advanced(800)	118(14.8 %)	682(85.2 %)	
CA125 Level	<500 U/ml(193)	15(7.8 %)	178(92.2 %)	0.004
	≥500 U/ml(661)	103(15.6 %)	558(84.4 %)	
Hyperfibrinogenemia	Yes (332)	77(23.2 %)	255(76.8 %)	<0.001
	No (391)	15(3.8 %)	376(96.2 %)	
Ascites^a^	No (99)	3(3.0 %)	96(97.0 %)	<0.001
	<500 ml(146)	5(3.4 %)	141(96.6 %)	
	≥500 ml(628)	113(18.0 %)	515(82.0 %)	
Cytoreduction^b^	R0(271)	18(6.6 %)	253(93.4 %)	<0.001
	R1(434)	67(15.4 %)	367(84.6 %)	
	R2(169)	36(21.3 %)	133(78.7 %)	
Chemosensitivity	Yes (567)	69(12.2 %)	498(87.8 %)	0.043
	No (237)	42(17.7 %)	195(82.3 %)	

^a^Subgroup analyses: P_No *vs <*500ml_ = 1.000, P_No vs >500ml_ < 0.001, P_<500 ml vs <500ml_ < 0.001

^b^Subgroup analyses: P_R0 *vs* R1_ = 0.001, P_R0 vs R2_ < 0.001, P_R1 vs R2_ = 0.092

**Table 3:** Clinicopathologic parameters of the patients and hyperfibrinogenemia

Parameters		Hyperfibrinogenemia		*P*-value
		Yes	No	
Age	<56(362)	177(48.9 %)	185(51.1 %)	0.117
	≥56(362)	155(42.8 %)	207(57.2 %)	
FIGO Stage	Early(62)	12(19.4 %)	50(80.6 %)	<0.001
	Advanced(662)	320(48.3 %)	342(51.7 %)	
CA125 Level	<500U/ml(157)	53(33.8 %)	104(66.2 %)	0.001
	≥500U/ml(556)	272(48.9 %)	284(51.1 %)	
Ascites^a^	No(85)	21(24.7 %)	64(75.3 %)	<0.001
	<500 ml(122)	22(18.0 %)	100(82.0 %)	
	≥500 ml(723)	289(56.0 %)	227(44.0 %)	
Cytoreduction^b^	R0(231)	70(30.3 %)	161(69.7 %)	<0.001
	R1(358)	179(50.0 %)	179(50.0 %)	
	R2(135)	83(61.5 %)	52(38.5 %)	
Chemosensitivity	Yes(474)	186(39.2 %)	288(60.8 %)	<0.001
	No(197)	118(59.5 %)	79(40.1 %)	

^a^Subgroup analyses: P_No *vs <*500ml_ = 0.296, P_No vs >500ml_ < 0.001, P_<500 ml vs <500ml_ < 0.001

^b^Subgroup analyses: P_R0 *vs R1*_ < 0.001, P_R0 vs R2_ < 0.001, P_R1 vs R2_ = 0.026

### Treatment outcomes, survival analysis, and their correlations with thrombocytosis and hyperfibrinogenemia

After primary surgery, 272 (31.1 %) of the patients were debulked to R0, and 434 (49.6 %) were debulked to <1 cm with macroscopic disease (R1). As the extent of residual disease increased, the numbers of patients who presented with thrombocytosis (R0, R1, and R2: 6.6 %, 15.4 %, 21.3 %, respectively, *p* < 0.001) and hyperfibrinogenemia (30.3 %, 50.0 %, 61.5 %, respectively, *p* < 0.001) increased. Pairwise comparisons revealed significant differences in the prevalences of hyperfibrinogenemia between the R0, R1 and R2 groups (R0 *vs* R1: *p* < 0.001, R0 *vs* R2: *p* < 0.001, R1 *vs* R2: *p* = 0.026). Regarding the platelet counts, a smaller proportion of the patients in the R0 group had thrombocytosis compared with the R1 and R2 groups (R0 *vs* R1: *p* = 0.001 and R0 *vs* R2: *p* < 0.001). However, the difference in the prevalence of thrombocytosis between the R1 and R2 groups was not significant (*p* =0.092).

Following primary surgery, 849 (97.0 %) patients had received platinum-based adjuvant chemotherapy. Among these patients, 568 (66.9 %) were chemosensitive (Table 1). Greater proportions of the patients with chemoresistant disease had documented thrombocytosis (17.7 % *vs* 12.2 %, *p* = 0.043) and hyperfibrinogenemia (59.5 % *vs* 39.2 %, *p* < 0.001) compared with the chemosensitive patients. Next, we investigated the correlations of chemosensitivity with thrombocytosis and hyperfibrinogenemia after stratification according to surgical outcome. The prevalence of thrombocytosis was no longer associated with chemosensitivity in the R0, R1 or R2 subgroups (p_R0_ = 0.744, p_R1_ = 0.660, and p_R2_ = 0.228). Regarding the fibrinogen levels, greater proportions of chemosensitive patients in the R0 and R1 groups had hyperfibrinogenemia compared with the corresponding chemoresistant patients (p_R0_ = 0.001 and p_R1_ = 0.004). However, the difference in hyperfibrinogenemia between the chemosensitive and chemoresistant patients was not significant in the R2 group (*p* =1.000).

The median(range) follow-up time was 29 (1–115) months. One hundred four (11.9 %) women experienced disease progression during adjuvant chemotherapy, 499 (57.0 %) patients exhibited documented recurrence, and 345 (39.4 %) deaths were documented. The median (95 % CI) PFS was 18 (16.8–19.2) months, and the 2-year and 5-year PFSs were 38.1 and 19.4 %, respectively. The median (95 % CI) OS was 58 (51.4–64.6) months, and the 2-year and 5-year OSs were 79.3 and 48.8 %, respectively.

Regarding PFS and OS, the known negative influences of advanced FIGO stage (*p* < 0.001 and <0.001, respectively), the presence of residual disease (R0 *vs* R1 + R2: *p* < 0.001 and <0.001, respectively), and chemoresistance (*p* < 0.001 and <0.001, respectively) were confirmed in the univariate analyses.

**Fig. 1:**
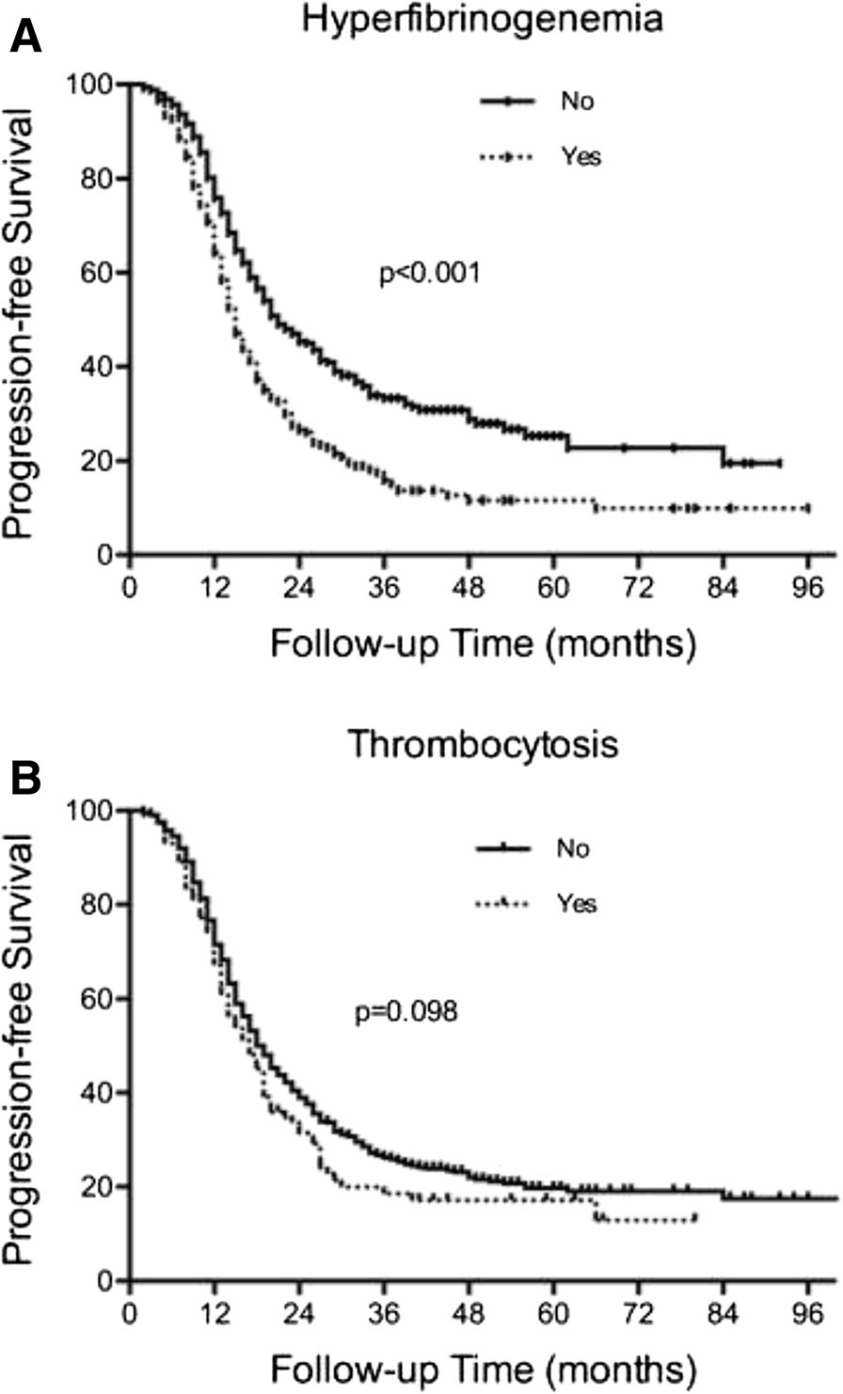
Kaplan-Meier curve of PFS stratified by hyperfibrinogenemia and thrombocytosis. (**a**) Preoperative hyperfibrinogenemia was associated with impaired PFS (*p* < 0.001). (**b**) The difference of PFS between patients with and without thrombocytosis was not significant (*p*=0.098)

In the univariate analysis, preoperative hyperfibrinogenemia was associated with impaired PFS (15.0 (13.8–16.2) *vs* 21.0 (17.8–24.2) months, *p* < 0.001, Fig. 1a). The women with thrombocytosis tended to exhibit shorter PFSs than those with normal platelet counts; however, this difference was not significant (17.0 (14.1–19.9) *vs* 19.0 (17.5–20.5) months, respectively, *p*=0.098; Fig. 1b). In the multivariate analysis with adjustments for age, FIGO stage, cytoreduction outcome and chemosensitivity status, preoperative hyperfibrinogenemia was not found to be independent predictor of PFS (HR = 1.202, 95 % CI, 0.991–1.459, *p* = 0.062; Table 4).

**Table 4:** Multivariable analysis of the factors associated with PFS

Characteristics		HR	95 % CI			*P* value
Age (as a continuous variable)		1.006	0.996	-	1.016	0.263
FIGO Stage	Early	Referent				
	Advanced	2.162	1.367	-	3.417	0.001
Cytoreduction	R0	Referent				
	R1	1.266	1.001	-	1.603	0.049
	R2	1.407	1.047	-	1.890	0.023
Chemosensitivity	No	Referent				
	Yes	0.083	0.065	-	0.107	<0.001
Hyperfibrinogenemia	No	Referent				
	Yes	1.202	0.991	-	1.459	0.062

**Fig. 2:**
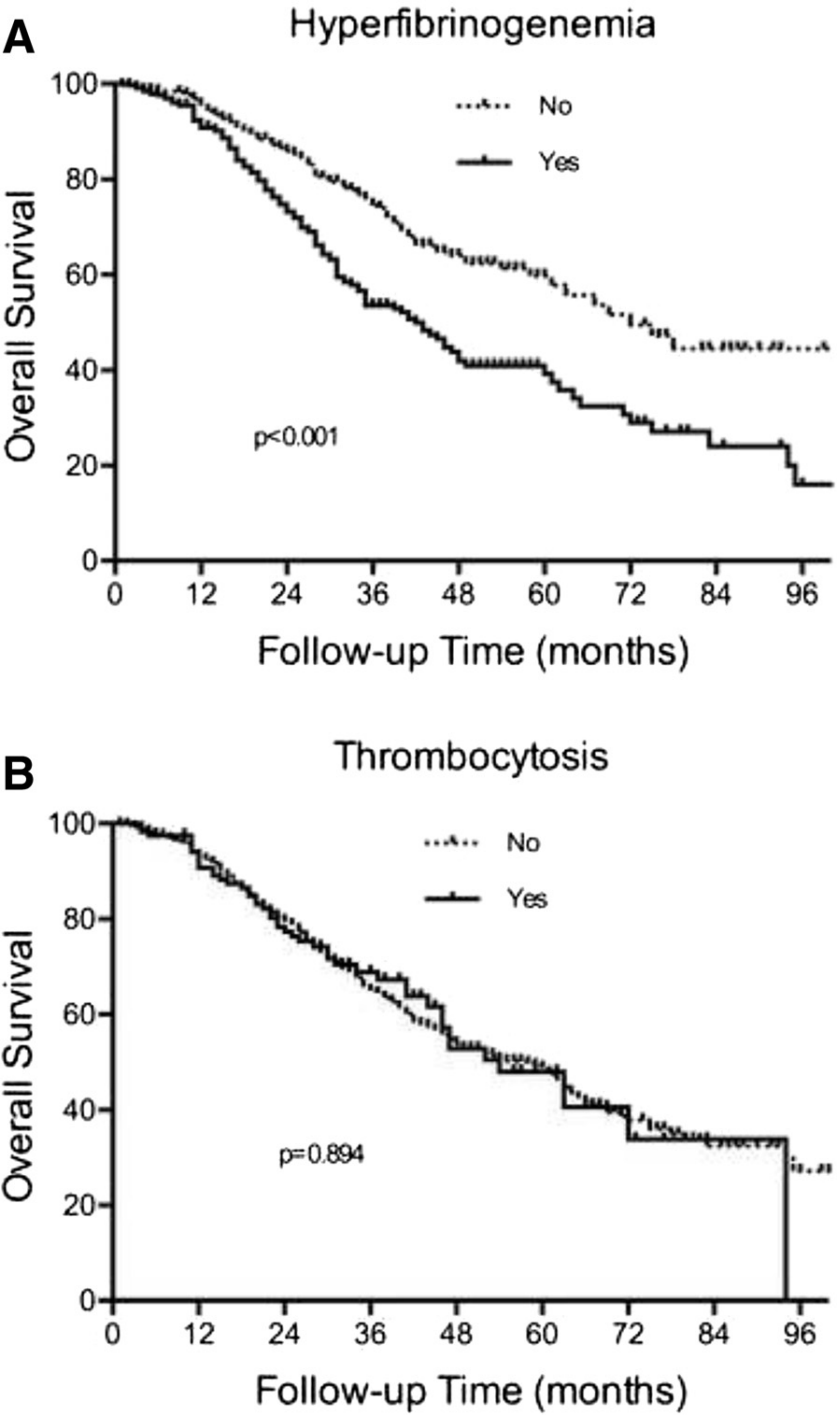
Kaplan-Meier curve of OS stratified by hyperfibrinogenemia and thrombocytosis. (**a**) Hyperfibrinogenemia was associated with a shorter OS (*p* < 0.001). (**b**) Thrombocytosis was not correlated with OS (*p*=0.894)

Hyperfibrinogenemia was associated with a shorter OS (42 (35.2–48.8) *vs* 72 (58.5–85.5) months, *p* < 0.001) in the univariate analysis, but thrombocytosis was not correlated with OS (54.0 (41.0–67.0) *vs* 58.0 (50.6–65.4) months, *p* = 0.894; Fig. 2). In the multivariate analysis with adjustments for age, FIGO stage, cytoreduction outcome and chemosensitivity status, hyperfibrinogenemia was independently associated with a poorer OS (HR = 1.523, 95 % CI, 1.179–1.967, *p* = 0.001; Table 5).

**Table 5:** Multivariable analysis of the factors associated with OS

Characteristics		HR	95 % CI			*P* value
Age (as a continuous variable)		1.021	1.006	-	1.036	0.005
FIGO Stage	Early	Referent				
	Advanced	3.574	1.522	-	8.393	0.003
Cytoreduction	R0	Referent				
	R1	1.518	1.060	-	2.175	0.023
	R2	1.412	0.929	-	2.147	0.107
Chemosensitivity	No	Referent				
	Yes	0.193	0.146	-	0.255	<0.001
Hyperfibrinogenemia	No	Referent				
	Yes	1.413	1.074	-	1.859	0.014

## Discussion

In this large mono-institutional study, we demonstrated that both thrombocytosis and hyperfibrinogenemia were associated with clinicopathological characteristics and treatment outcomes. Additionally, hyperfibrinogenemia was correlated with poorer overall survival in the Chinese patients with HGSC.

We found that both thrombocytosis and hyperfibrinogenemia were associated with increased tumor stage, elevated CA125 level, and more extensive ascites. These finding are in accordance with the previous published data from patients with epithelial ovarian cancer regardless of histologic type [2–5, 11], indicating that the platelet and fibrinogen levels seem to increase in parallel with tumor progression and metastasis. Experimental studies have proven that a hypercoagulable status can be induced directly by the tumor cells themselves or indirectly by the tumor-associated cytokines that are produced by host macrophages or endothelial cells [2, 12, 13], and interleukin-6 (IL-6) has been found to be a key regulator of paraneoplastic thrombocytosis and hyperfibrinogenemia [2, 12–15]. Consequently, we believe that coagulation parameters could reflect tumor burden to some extent. Additionally, IL-6-targeted therapy has been found to be capable benefiting the treatment of ovarian cancer [2, 16, 17].

Because they were correlated with increased disease burden, platelet count and plasma fibrinogen level could be considered to be predictors of surgical outcomes [2–6, 16]. Ma et al. [4] found that epithelial ovarian cancer patients with thrombocytosis have a greater likelihood of suboptimal cytoreduction (*p* = 0.035). Another multicenter study reported that an elevated fibrinogen level is associated with the presence of increased postoperative residual tumor mass (*p* < 0.001), which might be due to the increased operative complexity that results from increased tumor burden. Our study confirmed that both thrombocytosis and hyperfibrinogenemia were correlated with more extensive post-surgical residual disease.

Additionally, we investigated the relationship between hypercoagulability and chemosensitivity in HGSC patients for the first time. We observed that more patients with chemoresistant disease had documented thrombocytosis and hyperfibrinogenemia compared with the chemosensitive patients. Previous studies have demonstrated that coagulation system components can promote tumor growth, angiogenesis, and metastasis and thus interact with cytotoxic drugs [12, 13, 18–21]. The response to chemotherapy can be influenced by surgical residual disease, and we have proven that hypercoagulability was associated with more extensive residual disease. Therefore, we investigated the correlation between hypercoagulability and chemosensitivity following stratification by post-surgical residual disease. We found that only hyperfibrinogenemia was associated with chemosensitivity in the R0 and R1 groups and that thrombocytosis was no longer a predictor of chemotherapy response.

Several previous studies have also shown that platelet count and fibrinogen level are potential prognostic indicators in ovarian cancer patients [2–6, 11]. Once activated, the coagulation system can directly or indirectly promote disease progression and metastasis and thus lead to poorer prognosis [13, 19–22]. Consequently, we confirmed that hyperfibrinogenemia was an independent predictor of poorer OS in HGSC patients; however, we failed to demonstrate the prognostic significance of thrombocytosis. Allensworth et al. [3] proved that thrombocytosis is an independent predictor of PFS but not OS; however, when these authors stratified the patients according to FIGO stage, they found that thrombocytosis was no longer an independent predictor of PFS in the advanced stage (HR = 1.31, 0.99–1.75). Perhaps because the overwhelming majority (91.4 %) of our patients were in the advanced stage, no differences according to thrombocytosis were observed in our study.

The limitation of our study is that it was a retrospective study that depended on accurate documentation, and thus the potential for recall bias existed. Additionally, our center is one of the tertiary referral hospitals in China, and over 90 % of patients in such hospitals have advanced disease. Previous studies have shown that other coagulation biomarkers, including D-dimer and von Willebrand factor, can also reflect disease burden and serve as prognostic predictors [7, 8]. However, these parameters are not routinely examined in our daily work.

Notwithstanding its limitation, this was a mono-institutional retrospective study that included a large number of HGSC patients. This study involved a group of homogenous patients with the same histology who were treated within 9 years and underwent similar surgical procedures and consistent platinum-based chemotherapy regimens. And this could increase the generalizability of our study findings.

## Conclusion

In conclusion, our study suggests that both preoperative thrombocytosis and hyperfibrinogenemia reflect tumor burden to some extent and thus are predictive factors of treatment outcomes and that fibrinogen level could be regarded as a prognostic predictor in HGSC patients.

## Abbreviations

HGSC: high-grade serous ovarian cancer
PFS: progression-free survival
OS: overall survival
HRs: hazard ratios
RD: residual disease
SD: standard deviations
IQRs: interquartile ranges
